# Scaffold-Based Tissue Engineering Strategies for Osteochondral Repair

**DOI:** 10.3389/fbioe.2021.812383

**Published:** 2022-01-11

**Authors:** Jiang-Nan Fu, Xing Wang, Meng Yang, You-Rong Chen, Ji-Ying Zhang, Rong-Hui Deng, Zi-Ning Zhang, Jia-Kuo Yu, Fu-Zhen Yuan

**Affiliations:** ^1^ Department of Sports Medicine, Peking University Third Hospital, Beijing, China; ^2^ Institute of Sports Medicine of Peking University, Beijing, China; ^3^ Beijing National Laboratory for Molecular Sciences, State Key Laboratory of Polymer Physics and Chemistry, Institute of Chemistry, Chinese Academy of Sciences, Beijing, China; ^4^ University of Chinese Academy of Sciences, Beijing, China

**Keywords:** osteochondral repair, scaffolds, fabrication, tissue engineering, biomaterials

## Abstract

Over centuries, several advances have been made in osteochondral (OC) tissue engineering to regenerate more biomimetic tissue. As an essential component of tissue engineering, scaffolds provide structural and functional support for cell growth and differentiation. Numerous scaffold types, such as porous, hydrogel, fibrous, microsphere, metal, composite and decellularized matrix, have been reported and evaluated for OC tissue regeneration *in vitro* and *in vivo*, with respective advantages and disadvantages. Unfortunately, due to the inherent complexity of organizational structure and the objective limitations of manufacturing technologies and biomaterials, we have not yet achieved stable and satisfactory effects of OC defects repair. In this review, we summarize the complicated gradients of natural OC tissue and then discuss various osteochondral tissue engineering strategies, focusing on scaffold design with abundant cell resources, material types, fabrication techniques and functional properties.

## Introduction

The management and repair of osteochondral (OC) defects are still one of the most challenging clinical issues in orthopedics. Resulting from trauma, athletic injury or pathological factors, early localized osteochondral lesions can lead to general tissue deterioration, characterized clinically by severe pain and functional incapacitation of the affected joints (Hunter and Bierma-Zeinstra, 2019). As a common degenerative disease worldwide with high socioeconomic burdens, osteoarthritis (OA) is an adverse outcome of OC defects (Kwon et al., 2019). At the same time, OA can exacerbate the defects as a major cause. By 2030, approximately 67 millions people are expected to suffer from OA in the United States (Murphy and Helmick, 2012; Zhao et al., 2019). The upper articular cartilage possesses a stratified structure with no lymphatic or vascular components, lacking the capability of self-rehabilitation (Le et al., 2021). Moreover, different gradients of OC tissue have heterogeneous microstructures and biological properties (Ansari et al., 2019). So far, various clinical treatments have been available to alleviate symptoms and improve life quality to some extent, including microfracture technology, mosaicplasty, subchondral drilling, chondral shaving, abrasion arthroplasty, auto/allografts and joint replacement surgery (Redman et al., 2005; Gracitelli et al., 2016). Unfortunately, on account of the complex condition involving different layers of articular cartilage, cartilage-bone interface and subchondral bone, all these approaches have failed to achieve complete repair of OC defects and satisfactory reconstruction of joint functions. Then, the emergence of tissue engineering strategies has shown promise as a potential alternative for OC defect repair (Smith and Grande, 2015). This review aims to update the recent development in OC tissue engineering, focusing on biomaterial design and scaffold modification.

## Native Osteochondral Tissue: Structure and Tissue Engineering

### Articular Cartilage

**FIGURE 1:**
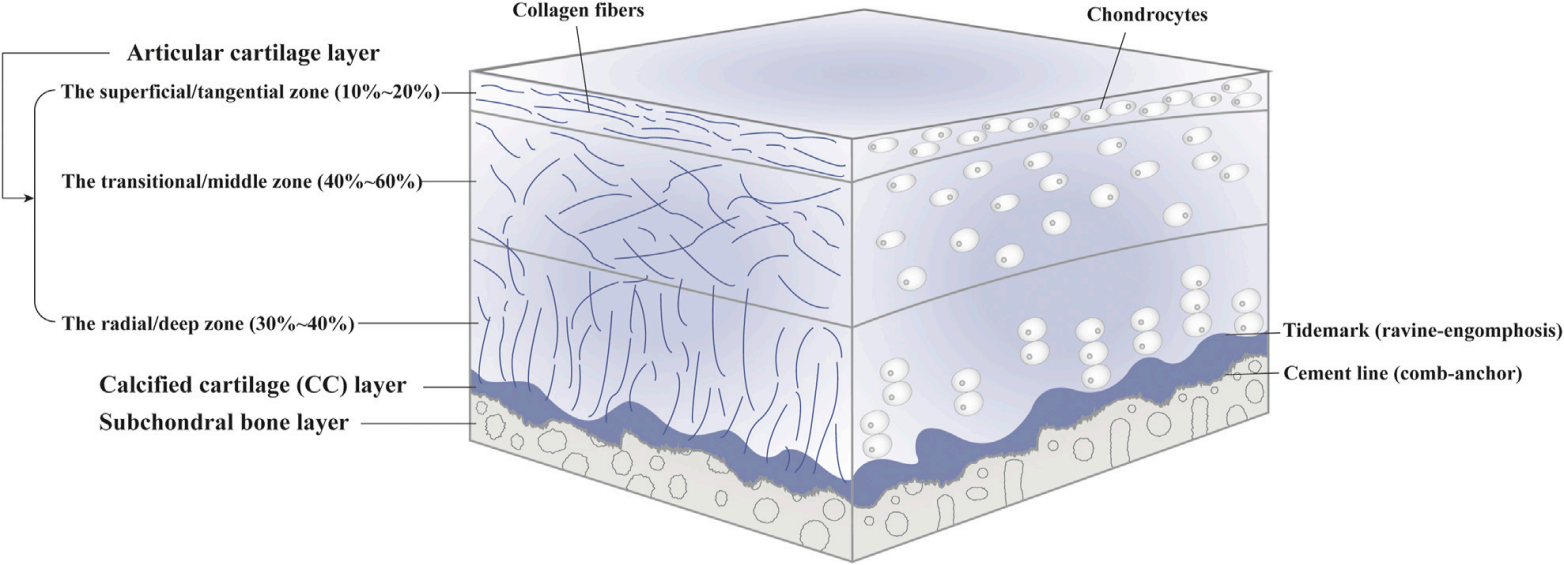
The components of native osteochondral (OC) tissue.

As a tough and flexible connective tissue lack of lymphatics, blood vessels and nerves, articular cartilage can be further divided into the radial/deep zone, the transitional/middle zone and the superficial/tangential zone, consisting of embedded chondrocytes and extracellular matrix (ECM) (Hunziker et al., 2002; Sophia Fox et al., 2009) (Figure 1). As the singular cell type, chondrocytes are responsible for the synthesis, homeostasis and remodeling of ECM. Also, they can sense local environment by expressing integrins (Loeser, 2014). Each chondrocyte and the surrounded narrow pericellular matrix (PCM) is referred to as a chondron (Poole et al., 1987). With tensile strength and unique viscoelastic properties, articular cartilage facilitates load transmission to subchondral bone during compression and restores original appearance when the pressure is relieved, performed by the fibrillar collagen network and entrapped macromolecules such as collagen II and proteoglycans in the ECM (Carballo et al., 2017). In addition, articular cartilage provides a lubricated surface to reduce friction with the presence of lubricin and hyaluronic acid. In terms of nutritional supplies, on the one hand, joint movement and mechanical stimulation cause synovial fluid to flow over the cartilage surface. On the other hand, small molecules can penetrate from subchondral bone into articular cartilage through potential direct signaling pathways (Pan et al., 2009). Signals associated with cartilage injury or scaffold implantation can recruit and activate immune cells, followed by cellular polarization. The phenotypes of cells and their interactions can affect the local microenvironment. Pro-regenerative microenvironment can develop proper tissue resembling the original host tissue; however, unbalanced immune system can produce inflammation and fibrocartilage, causing functional impairment (Sadtler et al., 2016).

### Calcified Cartilage

The calcified cartilage (CC) layer is defined as mineralized cartilage between articular cartilage layer and subchondral bone plate which passages from the so-called tidemark to the cement line (Ferguson et al., 2003; Lyons et al., 2006). The CC layer interlocked tightly with the upper articular cartilage and the lower subchondral bone plate in the manner of “ravine-engomphosis” and “comb-anchor”, respectively, (Wang et al., 2009) (Supplementary Figure S1). The undulated interface helps convert shear into compressive and tensile forces. Also, it can provide an integration to transfer mechanical load between flexible cartilage and stiff subchondral bone and maintain interfacial environment (Goldring and Goldring, 2016).

### Subchondral Bone

Subchondral bone refers to the bony layer beneath the cement line, which can be anatomically divided into subchondral bone plate (SBP) and trabecular bone (STB) (Goldring and Goldring, 2010). SBP is impenetrable cortical lamellae, whereas STB is more porous and metabolically active with lower volume, density and stiffness. Osteocytes, the most widely distributed cell throughout bone tissue, are involved in bone metabolism and mechanical transduction through solid matrix directly or load-induced fluid flow indirectly (Knothe Tate et al., 2004; Furuya et al., 2018). Collagen I accounts for over 90% of bone matrix (Blair et al., 2017; Schlesinger et al., 2020). The environment in subchondral bone has an effect on the viscoelasticity and nutritional metabolism of articular cartilage (Burr and Gallant, 2012; Fell et al., 2019; Hu et al., 2021).

### Gradients of the OC Tissue

**FIGURE 2:**
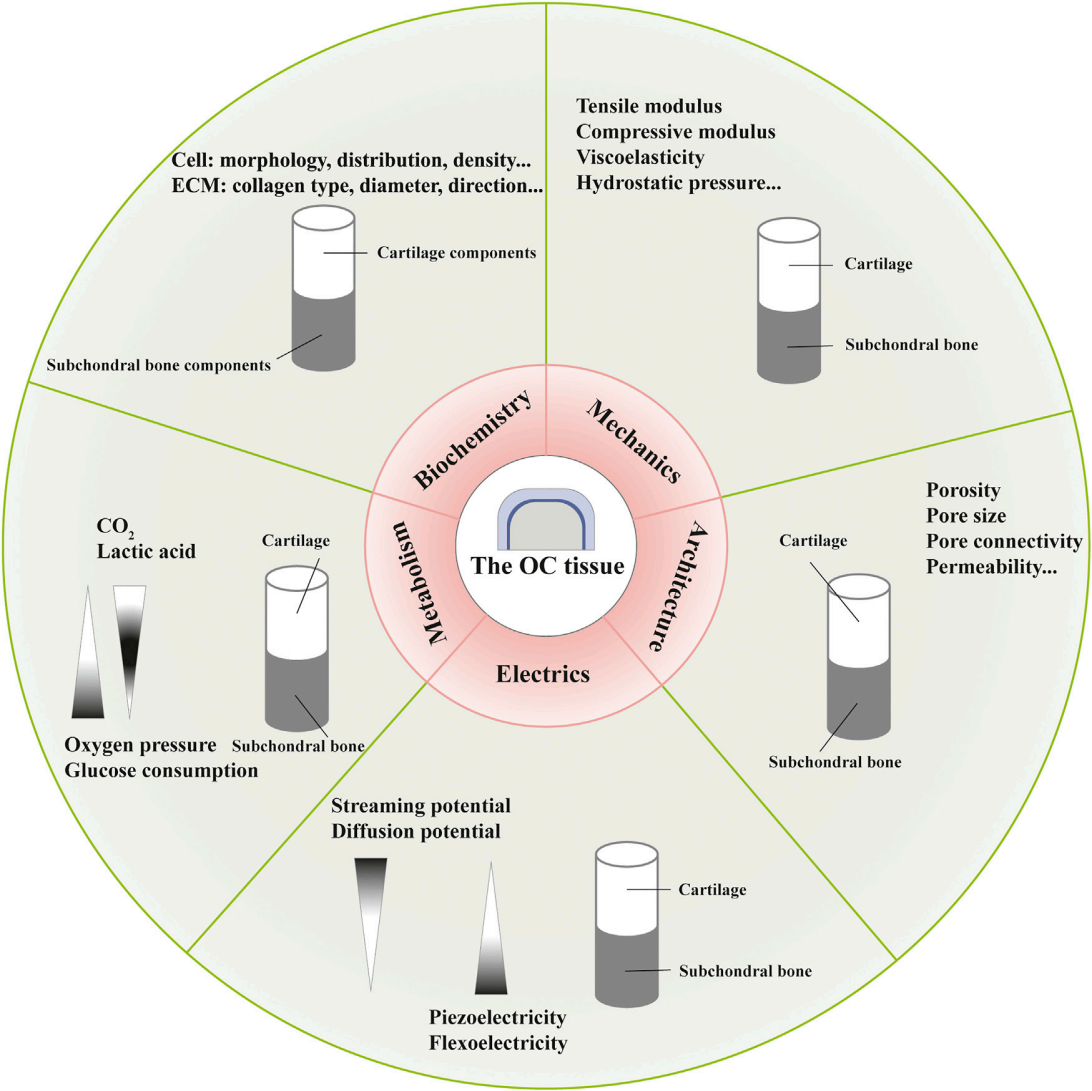
Different gradients in the osteochondral (OC) tissue with regard to biochemistry, mechanics, architecture, electrics and metabolism.

Distinct gradients and properties during development and maturation, with regard to biochemistry, mechanics, architecture, electrics and metabolism, have been found in the OC tissue, which are not completely independent and act as the foundation for OC functional tissue engineering (Ansari et al., 2019; Li et al., 2021) (Figure 2)*.*

The OC tissue exhibits cellular and compositional transitions from articular cartilage layer to subchondral bone layer (Figure 1). Within the articular cartilage layer, gradients of cell morphology, distribution and surrounding ECM can be observed (Ren et al., 2016). In the superficial/tangential zone, the flattened chondrocytes with the maximum cell density and highly packed collagen fibers aligned parallel to the articular surface, endowing the cartilage with tensile properties. As an anatomic and functional bridge between the superficial and radial zones, the transitional/middle zone contains more rounded cells at low density and thicker collagen fibers which are organized obliquely, functioning as the first line of resistance to compressive forces. Chondrocytes in the radial/deep zone are ellipsoid or rounded, arranged in columns perpendicular to the joint surface. Also, collagen fibers in this zone are organized vertical to the surface. In the CC layer, we can see sparce chondrocytes with hypertrophic types which can produce collagen X. The underlying bone tissue comprises a variety of cells such as osteoblasts, osteoclasts, osteocytes, chondrocytes, endothelial cells and mesenchymal stem cells (MSCs) (Florencio-Silva et al., 2015). Moreover, hydroxyapatite increases gradually moving from articular cartilage to bone layer. The specific composition and content of different gradients should be considered when designing complicated layered constructs in OC tissue engineering. For instance, polymers with various water absorption capabilities can be used in combination to mimic moisture gradient. Different growth factors can be integrated to rehabilitate specific differentiation paths.

Essential constituents (collagen and proteoglycan, for instance) and mineralization degree lead to depth-dependent variations in mechanical properties including tensile modulus, compressive modulus, viscoelastic properties, and hydrostatic pressure (Responte et al., 2007; Chen et al., 2013; Armiento et al., 2018). Owing to the intrinsic material features of soft collagen fibers and rigid apatite crystals, and hierarchical arrangements more importantly, the bone layer develops diverse mechanical properties with regard to the loading direction as an anisotropic and viscoelastic material (Morgan et al., 2018). Accordingly, a collagen-apatite composite scaffold has been fabricated to restore bone-like hierarchical organization (Wingender et al., 2018). To repair OC tissue, metal alloys and ceramics have been used to mimic the strength and stiffness of subchondral layer, while polymers for the viscoelasticity of cartilage layer (Khorshidi and Karkhaneh, 2018). Fabrication techniques, such as electrospinning, can modify the mechanical properties of regenerated constructs by producing fibers with different diameters, components and structures. And bio-reactors can provided mechanical stimulation to mimic physiological conditions of OC tissue and control cell differentiation (Karkhaneh et al., 2014).

Architectural gradients refer to structural features such as porosity, pore size, pore connectivity and permeability. Articular cartilage has open and connected pores with a porosity of 60–85%, while the cortical bone and trabecular bone are 5–30% and 30–90% porous, respectively, (Mow et al., 1992; Di Luca et al., 2015). With permeability changing in a deformation, direction and location dependent manner, the articular cartilage inhibits fluid loss and promotes nutrition transmission (Maleki et al., 2020). And the permeability of bone tissue changes with the density of osteocytes and fabric parameters (Kreipke and Niebur, 2017). These architectural properties are closely related to cell migration and vascular in-growth in tissue regeneration.

At present, three theories exist about the electrical characteristics of the cartilage layer: streaming potential, diffusion potential and piezoelectricity (Schmidt-Rohlfing et al., 2002; More and Kapusetti, 2017; Farooqi et al., 2019). The bone tissue can also generate electricity under pressure possibly due to piezoelectricity of collagen and flexoelectricity of bone mineral (Vasquez-Sancho et al., 2018). Scaffolds produced by piezoelectric materials can provide electrical energy and trigger signaling pathways associated with cell morphology maintenance, gene expression and biological functions, associated with tissue repair (Jacob et al., 2018).

In terms of metabolism, different properties of tissues result from mediums of physical transport—synovial fluid and blood vessels, respectively. The metabolic gradients include oxygen pressure, glucose consumption and waste products, which are associated with chondrocyte phenotype, cellular functions and environmental homeostasis (Sheehy et al., 2012; Karner and Long, 2018; Sieber et al., 2020; Suzuki et al., 2020). Controlled oxygen releasing biomaterials, such as hyperoxide and fluorinated compounds, are potential candidate for oxygen gradient formation in OC tissue engineering (Camci-Unal et al., 2013). And well designed bio-reactors can provide nutrients and remove waste products (Hossain et al., 2014).

Undoubtedly, to design a suitable construct for the rehabilitation of detected OC tissue is based on comprehensive understanding of native structure and physiology. Necessarily, multi-layered scaffolds, which are synthesized respectively and assembled subsequently, can not form a smooth transition between different layers, probably resulting in unsatisfactory simulation of native tissue interface. Several methods, such as microfluidic system, centrifugation, core-shell and layer-by-layer deposition, have been used to mimic physical and chemical gradients of native osteochondral tissue (Cross et al., 2016; Ansari et al., 2019). Advances in bio-reactors and fabrication techniques pave the way for mimicking the complex microenvironment of OC tissue. And coinduction of mechanical and electrical gradients combined with stratified metabolic regulation of cells should be concerned in future studies.

## Cell Sources in Osteochondral Tissue Engineering

**FIGURE 3:**
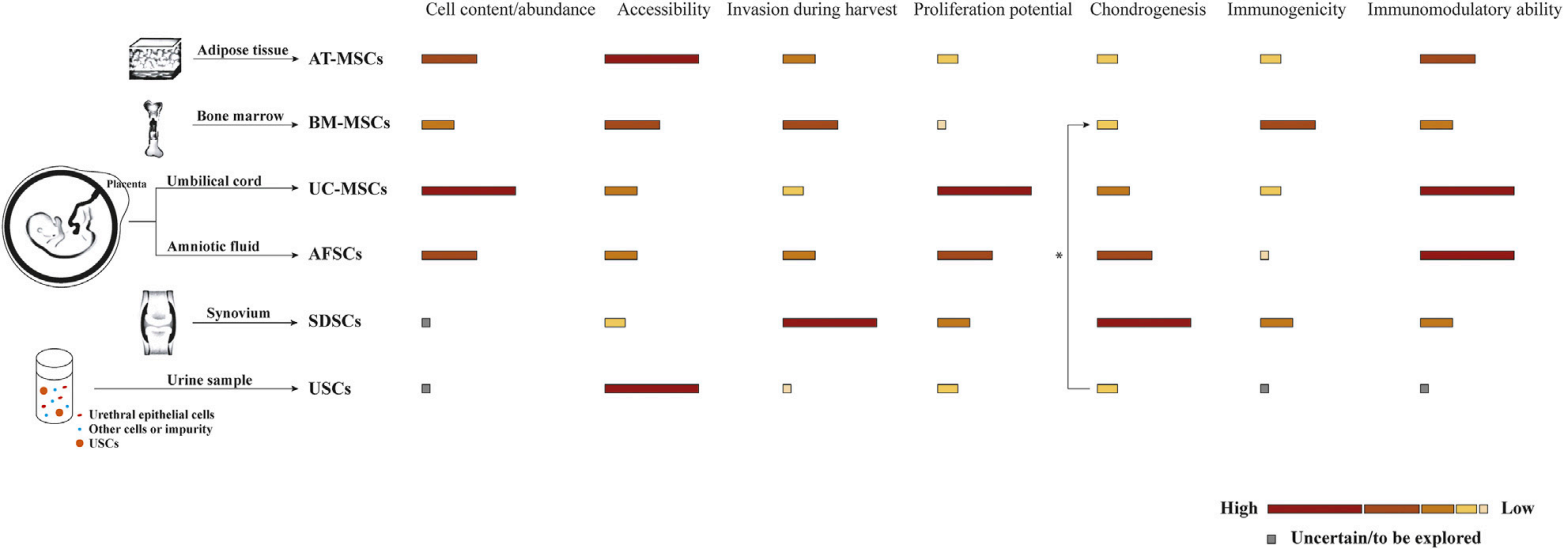
Comparison of stem cells from different sources commonly used in scaffold-based osteochondral (OC) tissue engineering strategies. Biological characteristics are shown as different color blocks from high to low, inspired by Zha et al. (2021). * USCs are reported to promote the chondrogenesis of BM-MSCs by paracrine action in the co-culture system (Gao et al., 2016).

In some OC tissue engineering strategies, scaffolds are preliminarily loaded with different cells to collectively promote tissue repair. Ideal cells for tissue engineering should have adequate sources and be able to maintain *in vitro* for manipulation and implantation safely. As shown in Table 1, two commonly proposed cell types for osteochondral repair are tissue-specific cells and progenitor cells, namely stem cells from different sources (Figure 3). Consistent with the host tissue, we usually use chondrocytes for hyaline cartilage repair and osteoblasts for subchondral bone regeneration. However, the use of differentiated cells suffers several limitations in the successive process of harvest, isolation, expansion, seeding, culture and finally implantation. For instance, chondrocytes are characterized by limited quantity in the native cartilage tissue, isolation difficulty and dedifferentiation capacity (Nukavarapu and Dorcemus, 2013). Since chondrocytes and osteoblasts both originate from bone marrow stem cells, MSCs have received widespread attention for their prominent advantages such as rapid proliferation and multipotency (Kagami et al., 2011; Mahmoudifar and Doran, 2013). Also, the secretory and immunomodulatory functions are closely related with cartilage regeneration. Various methods have been proposed for spatial and temporal control of differentiation toward the osteogenic and chondrogenic lineage including matrix properties and external factors, remaining to further explore and perfect (Seong et al., 2010; Mendes et al., 2018). Additionally, cell-free strategies have been pursued to overcome the aforementioned limitations (Maia et al., 2018). In combination with microfracture technology, biocompatible and biodegradable scaffolds without cells are implanted to promote cell recruitment and differentiation within the osteochondral defect area. Moreover, alternative approaches participated by MSC-derived exosomes or extracellular microvesicles have been developed in tissue repair and regeneration (Kim et al., 2020).

**TABLE 1:** Cell resources in osteochondral tissue engineering.

Cell types	Cell sources	Relevant characteristics
Tissue-specific cells	Chondrocytes	More functional cartilaginous tissue formation
		Limited quantity in the native tissue
		High integration into the surrounding matrix
		Dedifferentiation capacity during culture and expansion
	Osteoblasts	The expression of Runx2 peaks in immature osteoblasts and reduces at maturity Komori, (2019)
		Enhanced apoptosis by p53 and accelerated differentiation through Akt-FoxOs pathway Komori, (2016)
		Osteoblast-derived VEGF promotes bone repair and homeostasis Hu and Olsen, (2016)
Progenitor cells	BM-MSCs	Most widely used, but highly invasive
		The frequency, proliferation efficiency and differentiation potential decline with age
		Immunomodulatory functions, facilitating better tissue survival *in vivo* Sun et al. (2018); Ding et al. (2016)
	UC-MSCs	Inexhaustible supply, noninvasive procurement and high purity
		Faster proliferation rates, greater expansion capability and broad multipotency Baksh et al. (2007); Chen et al. (2009)
		More primitive—expressing both MSC and ESC markers Barrett et al. (2019)
		No or only mild immune response based on recent evidence Prasanna et al. (2010); Liu et al. (2012)
	AT-MSCs	Increased osteogenic differentiation by allylamine modification Murata et al. (2020); Chaves et al. (2016)
		The deposition of chemical groups (e.g., NH2 and COOH) affects chondrogenic and osteogenic lineages Griffin et al. (2017)
	SDSCs	Better proliferation and chondrogenic differentiation performance than BM-MSCs and AT-MSCs Sasaki et al. (2018); Zheng et al. (2015)
		Weaker osteogenic capability than BM-MSCs
		Elevated ECM deposition and inhibited hypertrophy of chondrocytes Kim et al. (2018)
	AFSCs	Expressing Runx2, osterix, osteopontin et al. and producing extracellular calcium stores during differentiation Maraldi et al. (2011)
		Typical differentiation process into cells of mesodermal origin regulated by growth factors (e.g., TGF-β, IGF-1 and EGF) Bajek et al. (2014)
	USCs	A recently reported candidate for seed cells in tissue engineering Gao et al. (2016)
		Osteogenic and chondrogenic potentials worth exploring Qin et al. (2014)
		Simple isolation and culture, non-invasive and easy obtainment, low-cost and high efficiency Zhang et al. (2008); Guan et al. (2014)

Abbreviations: BM-MSCs, Bone marrow-derived MSCs; UC-MSCs, Umbilical cord MSCs; AT-MSCs, Adipose tissue-derived MSCs; SDSCs, Synovium-derived MSCs; AFSCs, Amniotic fluid-derived stem cells; USCs, Urine-derived stem cells.

## Scaffold Fabrication Techniques

Scaffolds are one of the fundamental elements of tissue engineering approaches to osteochondral repair. For the quality of tissue regeneration, scaffolds are expected to have the following characteristics: porous structure for cell survival and material transport, suitable surface for cell adhesion, proliferation and differentiation, mechanical properties matching the surrounding tissue, biocompatibility with limited immunoreaction and bioabsorbability with a controllable degradation rate (Hutmacher, 2000).

To improve scalability, sustainability and spatial control, various methods for scaffold fabrication in osteochondral tissue engineering have been proposed, including lyophilization, freeze casting, gas foaming, sol-gel process, solvent casting, compression molding, particulate leaching and phase separation process et al. (Hutmacher, 2000; Cheng et al., 2019) (Table 2)*.* Seo JP et al. utilized the freeze-drying technology to prepare bilayer gelatin/β-tricalcium phosphate (GT) sponges (Seo et al., 2013). In their study, PRP/BMP-2/GT scaffolds showed more cartilage-like tissue with no remaining implant materials and no evidence of infection, adhesions or synoyial proliferation. To establish various bioactive scaffolds, the use of freeze casting has drawn much concern in recent years. In a previous study, Abarrategi A et al. performed both *in vitro* and *in vivo* assays of cell survival and bone formation based on rhBMP-2/multiwall carbon nanotubes (MWCNT)/chitosan (CHI) scaffolds in conjunction with freeze casting (Abarrategi et al., 2008). The technology has high application value and development potential in forming structures in accordance with natural tissue (Shao et al., 2020). Reyes R et al. analyzed the effects of repair induced by TGF-β1/BMP-2 loaded segmented polyurethane/polylactic-co-glycolic (SPU/PLGA) scaffolds in osteochondral lesions (Reyes et al., 2014). The PLGA porous structure was produced by gas foaming in acidic aqueous solution. Algul D et al. manufactured multilayered β-TCP/chitosan-alginate polyelectrolyte complex (CA/PEC) scaffolds to mimic the structural gradients of native osteochondral tissue (Algul et al., 2015). The chitosan and alginate solutions were mixed and stirred to prepare a gel. The chitosan/alginate gel was treated through a series of steps for scaffold fabrication such as lyophilization, cross-linking and elution. Wu et al. evaluated the efficacy of bilayered silk scaffolds loaded with TGF-β3/BMP-2 for osteochondral defect repair in rabbits aided by the solvent casting/particulate leaching technology (Wu et al., 2021). The lyophilized silk powder was first mixed with hexafluoroisopropanol (HFIP) solution with or without sucrose particles in a silicone mold. Then, 10% silk-HFIP solution was poured onto the mold and kept for crosslink. The demolded scaffolds were treated with methanol (100% w/v) and running water to remove the HFIP and particles. After the process above, the top and bottom layers of scaffolds served as the cartilage and subchondral bone layers, respectively. Moreover, Sil-MA (methacrylated silk fibroin) hydrogels for marginal sealing were prepared by photo curing. This new approach indicated the effect of the marginal sealant on the integration of the cartilage layer and rapid cartilage formation. Duan et al. investigated the effects of pore size on osteochondral repair *in vivo* using a rabbit model (Duan et al., 2014). The bilayered poly(lactide-co-glycolide) (PLGA) porous scaffolds for research were fabricated by a compression molding/particulate leaching method. The PLGA solution and sodium chloride (NaCl) particulates as porogen were mixed and pressed into the pre-designed mold. After releasing the mold, cylindrical structure was obtained. Then, the mixture constructs were cut into appropriate sizes and leached by water. Five types of integrated scaffolds with identical porosity and different pore sizes were processed finally. The assessment in this study reminded us to take pore sizes into consideration during scaffold design for tissue engineering. Da et al. prepared the compact layer between the chondral and bony layers from PLGA/β-TCP by the phase separation process (Da et al., 2013). The homogeneous PLGA/β-TCP mixture was compactly extruded and solidified line by line above the subchondral bone layer. Through *in vitro* and *in vivo* tests, the compact layer-containing biphasic scaffolds showed better biomechanical properties and tissue repair results.

**TABLE 2:** Fabrication techniques of scaffolds in osteochondral tissue engineering.

Techniques	Processes	The pros and cons
Lyophilization	The mixture is cooled by freeze-drying to eliminate the solvent and water, forming macropores and micropores in the scaffold structure	• The pore size and porosity can be modified by solution characteristics (e.g., concentration and viscosity), quenching rate and freezing temperature (Tf). Raeisdasteh Hokmabad et al. (2017)
		• The use of organic solvents; instability of the emulsion
Freeze casting	The manufacturing technique includes the controlled solidification process, the sublimation of solvents under reduced pressure and subsequent densification	• The applicability to various materials; changeable micro- and macrostructures of obtained scaffolds
Gas foaming	The raw materials are kept under a high carbon dioxide pressure to produce porous structures	• The uniformity of cell infiltration should be improved. Salonius et al. (2019)
Microfluidic foaming	The foam is generated via microfluidics under highly controlled and reproducible conditions	• Homogeneous pore monodispersity and interconnection; abundant cell infiltration; versatility. Costantini et al. (2016)
		• There is still room to expand the range of applicable biomaterials
Sol-gel process	The sol-gel method can result in oxides or hybrid materials in soft conditions	• Combined with other techniques, such as 3D printing, this approach can open a new way for the design of biocompatible hydrogels by promoting cross-linking. Valot et al. (2019); Tourné-Péteilh et al. (2019); Raucci et al. (2018)
Solvent casting	The polymer solution is first combined with necessary particles and then poured onto pre-designed molds	• Addition of functional elements such as drugs and growth factors
		• The potential toxicity of organic solvents
Melt molding	The mixture of powdered polymers and porogen is loaded into pre-designed molds and annealed at an elevated pressure	• Porous scaffolds with desired morphological features
		• The difficulty of later particulate leaching; high processing temperature; inapplicability of organic solvents
Compression molding	The mixture is pressed into molds under heat and pressure to obtain the required structures. Sempertegui et al. (2018); Zhang et al. (2016)	• High-pressure molding can compact the stacking structure and optimize mechanical performance
Particulate leaching	The preliminarily obtained scaffolds are treated and soaked to leach out particles	• Porous structures adjusted by the added porogen as required
		• The technical demands for better control of pore morphology and interconnection; extra time consumption
Phase separation process	The polymer solution is quenched under the freezing point (Tk) and separated into a polymer-rich phase and a polymer-poor phase which will solidify and crystallize respectively. Crystals are removed subsequently	• The scaffold structure can be tunable on account of processing parameters such as quenching temperature and rate
		• The improvement and integration of techniques is needed to optimize the probably unfavorable pore structure
Electrospinning	Under a strong electric field, a polymer solution, emulsion or melt is extruded through a spinneret to produce fibre and deposit on an appropriate collector	• Structures resembling the native ECM; encapsulation of bioactive elements
		• Poor control over architectures restricted by environmental parameters; difficulty in producing 3D structures; limited cell passage and substance exchange related to pore size; environmental safety issues
Additive manufacturing (AM)	The electrohydrodynamic technique, also known as rapid prototyping or solid freeform fabrication, is classified into seven processes: vat photopolymerization, material jetting, material extrusion, powder bed fusion, directed energy deposition, sheet lamination and binder jetting. Tang et al. (2016); Gibbs et al. (2014)	• Better control over architectures; flexibility to scale-up customisation; standardisation and repeatability of manufacturing
		• Narrow range of suitable materials, time-consuming layer-by-layer processing and high costs

**FIGURE 4:**
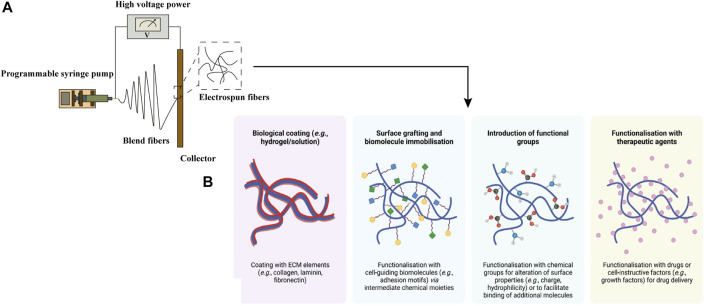
Electrospun fibers, obtained by basic electrospinning setup **(A)**, can be modified by physical or chemical techniques. Post-fabrication surface modifications of electrospun fibers **(B)** were adapted with permission from ref (Gonçalves et al., 2021). Copyright 2021 by MDPI Inc.

With the development of manufacturing, an advanced technique named electrospinning has been applied to produce functional scaffolds with spatially complex physical and chemical properties in osteochondral tissue engineering (Figure 4). To reduce the inflammatory immune responses, natural polymers such as collagen and silk fibroin have been employed as scaffold materials. Liu et al. fabricated a nanofiber yarn-collagen type I/hyaluronate hybrid (Yarn-CH)/TCP biphasic scaffold by dynamic liquid electrospinning, which showed an almost smooth articulating surface and good integrity of the host-implant interface (Liu S et al., 2015). Composite electrospun matrix derived from 70S bioactive glass and silk fibroin was obtained and evaluated as potential candidate for osteochondral defect repair by M JC et al. (M et al., 2017). The biphasic constructs showed the ability to synergistically support the chondrogenic and osteogenic growth. Also, certain synthetic polymers have been used as bioassimilable materials, including PCL, PLA and PLGA in particular (Liu W et al., 2015). The nanofibers fabricated by electrospinning technique can present a high surface area for cell growth and cellular differentiation (Aguirre-Chagala et al., 2017). Continuously graded insulin/PCL/β-GP scaffolds, fabricated via the application of the twin-screw extrusion/electrospinning method, have implemented the selective differentiation of h-ADSCs into chondrocytes and mineralized tissue hierarchically (Erisken et al., 2011). In the study of Baumgartner W et al., electrospun meshes of PLGA/amorphous calcium phosphate (aCaP) seeded with h-ADSCs were cultured in DMEM to explore the effects of aCaP and shear forces on osteogenic, chondrogenic, adipogenic and angiogenic stimulation (Baumgartner et al., 2019). To improve the reparative potential of irregular osteochondral defects, the cell/nanofibers composite electrospun scaffolds with a slurry-like texture have been produced. Kim HS et al. prepared the cartilage-dECM-decorated PCL electrospun scaffolds which were surface-modified with poly(glycidyl methacrylate) (PGMA@NF) via surface-initiated atom transfer radical polymerization (SI-ATRP) method (Kim et al., 2021). The *in vivo* study in a rat model indicated significantly improved cartilage and bone regeneration of osteochondral defects. To provide molecular cues and improve the spatiotemporal control of cell distribution and differentiation, Liu YY et al. reported a functional gradient scaffold consisting of gelatin/sodium alginate (SA) struts and electrospun nanofibers incorporated by gentamycin sulfate (GS) and desferoxamine (DFO) (Liu et al., 2016). Fabricated via a system which combined 3D biological printing and electrospinning process, the scaffold showed excellent mechanical stability. Loaded with different biomolecules, the 3D composite osteochondral scaffolds can achieve spatiotemporal release according to various requirements.

Osteochondral scaffold manufacturing that can produce tunable structure in terms of architectural, biomechanical and biochemical properties is an ideal candidate for tissue engineering. Additive manufacturing (AM), an electrohydrodynamic technique, is gradually coming into focus. Characterized by layered sedimentation and computer-aided design/manufacturing (CAD/CAM) technology, AM methods include fused deposition modeling (FDM), selective laser sintering (SLS) and inkjet 3D printing et al. (Gonçalves et al., 2021). Ding C et al. designed a CAD/CAM-fabricated scaffold from polylactic acid-coated polyglycolic acid (PGA/PLA) and poly-ε-caprolactone/hydroxyapatite (PCL/HA) for goat femoral head regeneration (Ding et al., 2013). The PGA/PLA and PCL/HA scaffolds were respectively fabricated by 3D printing and FDM with aid of CAD/CAM technology. The strategy in this study provided a promising approach for tissue specific regeneration with cartilage tissue, immature calcified tissue, transitional trabecular bone and hypertrophic chondrocytes. Significantly, these methods can integrate bioactive molecules or drugs into scaffolds derived from various biomaterials. Tamjid E et al. prepared PCL composite scaffolds containing tetracycline hydrochloride (TCH) by 3D printing and tested the performance of biocompatibility, drug release kinetics and antibacterial activity (Tamjid et al., 2020). This study has paved the way for realizing sustainable release of loaded medicine. In another instance, an air-extrusion 3D printing technique was utilized to fabricate a scaffold consisting of poly(N-acryloyl 2-glycine)-methacrylated gelatin (PACG-GelMA)-Mn^2+^ for cartilage layer and PACG-GelMA-bioactive glass (BG) for bone layer (Gao et al., 2019). *In vitro* experiments indicated increased gene expression of both chondrogenic- and osteogenic-related differentiation. Also, the biohybrid scaffold promoted osteochondral tissue regeneration after implantation in a rat model. With excellent elasticity, hybrid scaffolds can support various deformation. In some cases, osteochondral defects are closely associated with mitochondrial dysfunction, metabolic reconfiguration and increased heterochromatin, which provides a potential avenue to design functional scaffolds for tissue regeneration (Varela-Eirin et al., 2018; Coryell et al., 2021). Chen P et al. examined the therapeutic potential of MSC-derived exosomes in 3D printed ECM/GelMA scaffolds (Chen et al., 2019). *In vitro* studies demonstrated increased chondrocyte migration by the scaffolds, which has been reported as a major element in the repair process of osteochondral defects (Chen et al., 2015; Zhang et al., 2018). The healing capacity was then assessed in a rabbit model. After implantation, the ECM/GelMA/exosome scaffold was shown to restore the damaged chondrocyte mitochondria by providing critical proteins and stimulate M2 macrophage polarization in the synovium. Advances in manufacturing have allowed the development of *in situ* 3D printing technology for osteochondral tissue repair, thereby improving the surgical procedure and the graft accuracy (Ma et al., 2020). However, mass industrial implementation and clinical applications are limited by several limitations: narrow range of suitable materials, time-consuming layer-by-layer processing and high costs, et al. Further exploration is needed to accelerate the clinical translation and fill the gap in osteochondral defect treatment.

## Different Types of Scaffolds in Osteochondral Tissue Engineering

As a vital component of tissue engineering and regenerative medicine, scaffolds play an increasingly important role in the reconstruction and maintenance of tissue functions. Under the regulation of essential parameters, such as growth factors and functional particles, they can provide a platform for cell survival, proliferation and differentiation, thereby raising the possibility of tissue repair. Today, advances in biomaterials and fabrication techniques provide new opportunities for the development of biomimetic and sophisticated scaffolds, which can be further divided into monophasic, biphasic and multiphasic constructs. Apparently, multiphasic scaffolds have several advantages in bionic performance over the monophasic ones. In the following sections, we focus on different types of biphasic and multiphasic scaffolds for OC tissue engineering on the basis of biomaterial design (Supplementary Table S1).

### Porous Scaffolds

**FIGURE 5:**
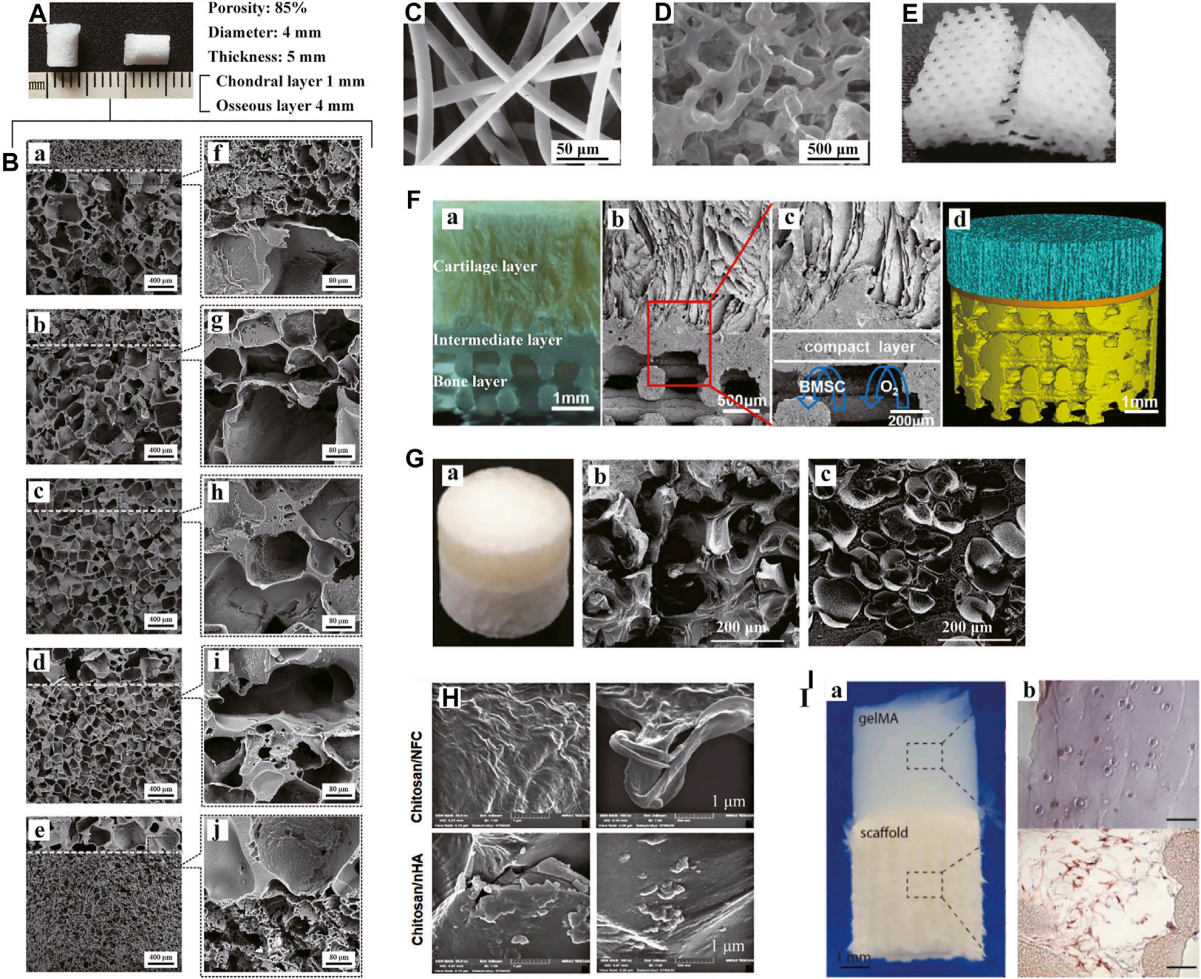
Porous and hydrogel scaffolds. Bilayered PLGA porous scaffolds **(A)** with different pore sizes shown in scanning electron microscopy (SEM) images **(B: a-e)**. The boundaries between the layers were magnified **(B: f-j)**. Bilayered porous scaffold consisting of non-woven PGA meshes **(C)** and PLGA/PEG foams **(D)**. PCL porous scaffold with a 0/60/120° lay-down pattern **(E)**. Muti-layered scaffolds with porous 3D printing PLGA/TCP bone layer **(F)** were shown in digital pictures **(F: a)**, SEM images **(F: b-c)** and micro-CT reconstructive images **(F: d)**, respectively. Bilayered CAN-PAC hydrogel **(G: a)** and the SEM images of upper **(G: b)** and lower layers **(G: c)**. SEM images of the upper and lower layers of chitosan hydrogel scaffold **(H)**. Tri-phasic scaffolds made of gelMA hydrogel and PCL/HA **(I: a)**. CD31 immunohistochemical analysis showed the presence of HUVECs in the PCL-PCL/HA phase **(I: b)**. **(A, B)** Adapted with permission from ref (Duan et al., 2014). Copyright 2013 by WILEY PERIODICALS Inc. **(C, D)** Adapted with permission from ref (Schaefer et al., 2000). Copyright 2000 by Elsevier Science Ltd. **(E)** Adapted with permission from ref (Cao et al., 2003). Copyright 2003 by Mary Ann Liebert Inc. **(F)** Adapted with permission from ref (Jia et al., 2018). Copyright 2018 by American Chemical Society. **(G)** Adapted with permission from ref (Liao et al., 2017). Copyright 2017 by Nature Publishing Group. **(H)** Adapted with permission from ref (Dehghani Nazhvani et al., 2021). Copyright 2021 by Elsevier Inc. **(I)** Adapted with permission from ref (Pirosa et al., 2021). Copyright 2021 by Elsevier Ltd.

There are various forms of porous scaffolds, including foam, sponge, mesh, microfibers and nanofibers, etc. The porous structure can function as an important support for cell aggregation and infiltration to guide further differentiation towards diverse lineages. Moreover, the interconnected pore networks with appropriate pore size and porosity are the forming basis of extracellular matrix simulating the native OC tissue. The resulting microenvironment can promote nutrient supply and stimulate effective cell-cell and cell-matrix communications. More significantly, the macro- and micro-pores of scaffolds are crucial channels for blood vessel and nerve growth. Considerable efforts have been made to optimize the structures of porous scaffolds to serve specific functions through tissue regeneration. As mentioned in the previous section, Duan P et al. first assessed the effects of pore size on the efficacy of *in vivo* osteochondral tissue repair (Figures 5A,B). Until now, researchers have not yet reached a consensus on the ideal pore morphology. In addition to pore size and porosity, more descriptive parameters (e.g., tortuosity and surface area to volume ratio, et al.) have been supplemented to characterize and evaluate scaffolds.

Ideal porous scaffolds for clinical applications in osteochondral tissue engineering should meet both structural and functional requirements. Different layers of the scaffolds can mimic the natural gradients of the OC tissue. After implantation, the scaffolds can stimulate tissue regeneration and integration with the surrounding cartilage and subchondral bone. Natural and synthetic polymers are commonly employed as biomaterials. In an earlier *in vitro* study, Schaefer D et al. dynamically seeded the fibrous, non-woven PGA meshes and PLGA/PEG foams with chondrocytes and periosteal cells, respectively, (Figures 5C,D). Then, the two cell-polymer constructs were cultured independently and sutured together for an additional co-culture in osteogenic medium. Mature bone-like constructs in combination with immature cartilaginous constructs were suggested to promote integration at the tissue interface. Seo JP et al. inserted PRP/acidic GT sponges loaded with chondrocytes and BMSCs and BMP-2/basic GT sponges loaded with BMSCs into the upper and lower part of osteochondral defects separately in a horse model (Seo et al., 2013). The GT sponges prepared have a porosity of 95.9% with the pore size of 179.1 ± 27.8 μm. Sponges have better mechanical stability compared to meshes. To date, there have been various multi-layered porous scaffolds reported based on different fabricating techniques. Dresing I et al. demonstrated PUR and nHA/PUR scaffolds consisting of three regions (Dresing et al., 2014). The scaffolds were assembled via a solvent welding technique. Jia S et al. designed a biomimetic multi-layered scaffold (Figures 5F). The scaffold comprised three integrated layers: an oriented ACECM-derived cartilage layer, an intermediate compact interfacial layer and a 3DP porous PLGA/TCP layer. The heterogeneous porous constructs provided a suitable template to guide hyaline cartilage, calcified cartilage and subchondral bone growth.

### Hydrogel Scaffolds

The extracellular matrix of osteochondral tissue is gelatinous substance containing fibrous components (Khorshidi and Karkhaneh, 2018). Because of the structural and compositional resemblance to natural ECM, natural and synthetic hydrogel scaffolds have great potential in tissue regeneration due to their intrinsic properties, such as biocompatibility, biodegrability and cell interaction. Natural materials used in hydrogels, including decellularized ECM, collagen, chitosan and hyaluronic acid etc., possess satisfactory biocompatibility with no inflammation and cytotoxity, whereas synthetic polymers are easier to process and control (Naahidi et al., 2017). To balance the degradability of scaffolds and the adhesions of cells is the key to design hydrogels for tissue repair. Responsive hydrogel materials are considered as potential candidate for biological platforms due to their adaptive responses to physiological stimuli in the environment. And hydrogel-based soft-hard interfaces, which can regulate homeostasis in the interfacial microenvironment, deserve attention in future studies (Fang et al., 2021).

A biphasic CAN-PAC hydrogel was fabricated by Liao J et al. for osteochondral defect regeneration in a rabbit model (Figures 5G). CSMA and NIPAm were dissolved in the upper solution, meanwhile, PECDA, AAm and PEGDA were added in the lower solution. The hydrogel facilitated the formation of new translucent cartilage and repaired subchondral bone. Also, a hypoxic preconditioned chitosan-based hydrogel has shown a faster healing trend, with thicker cartilage and more new ECM formation (Figures 5H). More recently, Pirosa A et al. used solid gelMA hydrogel in combination with wet-spun PCL and PCL/HA to generate an *in vitro* vascularized osteochondral tissue model (Figures 5I). In another study, an injectable and self-hardening hydrogel of silylated cellulose and chitosan showed the potential for osteochondral tissue repair in a dog model (Boyer et al., 2020).

### Fibrous Scaffolds

Actually, fibrous scaffolds can be classified as porous microfiber or nanofiber scaffolds, which offer a microenvironment favorable for cell attachment and survival. Various materials and techniques can be utilized for fabrications. Electrospinning, in particular, is an widely used technique. Moreover, the fibers can be specifically functionalized by the controlled release of drugs and biomolecules. The application of fibrous scaffolds to osteochondral tissue engineering makes it possible to customize properties as required, including pore morphology, resilience, flexibility and bioactivity etc.

In osteochondral tissue engineering, materials used to produce fibrous scaffolds include natural polymers, synthetic polymers, cellulose fibers, mineral fibers and carbon fibers (Hild et al., 2011; Yunos et al., 2013; Baah-Dwomoh et al., 2015). Liu S et al. produced a nanofiber yarn-collagen type I/hyaluronate hybrid (Yarn-CH)/TCP biphasic scaffold for osteochondral defect repair in a rabbit model. The nanofibrous scaffold showed an almost smooth articulating surface and good integration of cartilage-implant interface. A bilayered microporous scaffold was fabricated with collagen and electrospun poly-L-lactic acid nanofibers (COL-nanofibers) by Zhang S et al. The study reported more rapid osteogenic differentiation and better cartilage formation induced by the nanofibrous scaffold. Another nanofibrous scaffold, namely XanoMatrix, contains polyethylene terephthalate and cellulose acetate (Bhardwaj and Webster, 2016). With great hydrophobicity, 3D surface area and high tensile strength, the scaffold is a candidate for osteochondral defect treatment. Elangomannan S et al. evaluated the properties of carbon nanofibers/PCL/mineralized hydroxyapatite (CNF/PCL/M-HAP) scaffolds and reported the potential for orthopedic applications (Elangomannan et al., 2017).

### Microsphere Scaffolds

**FIGURE 6:**
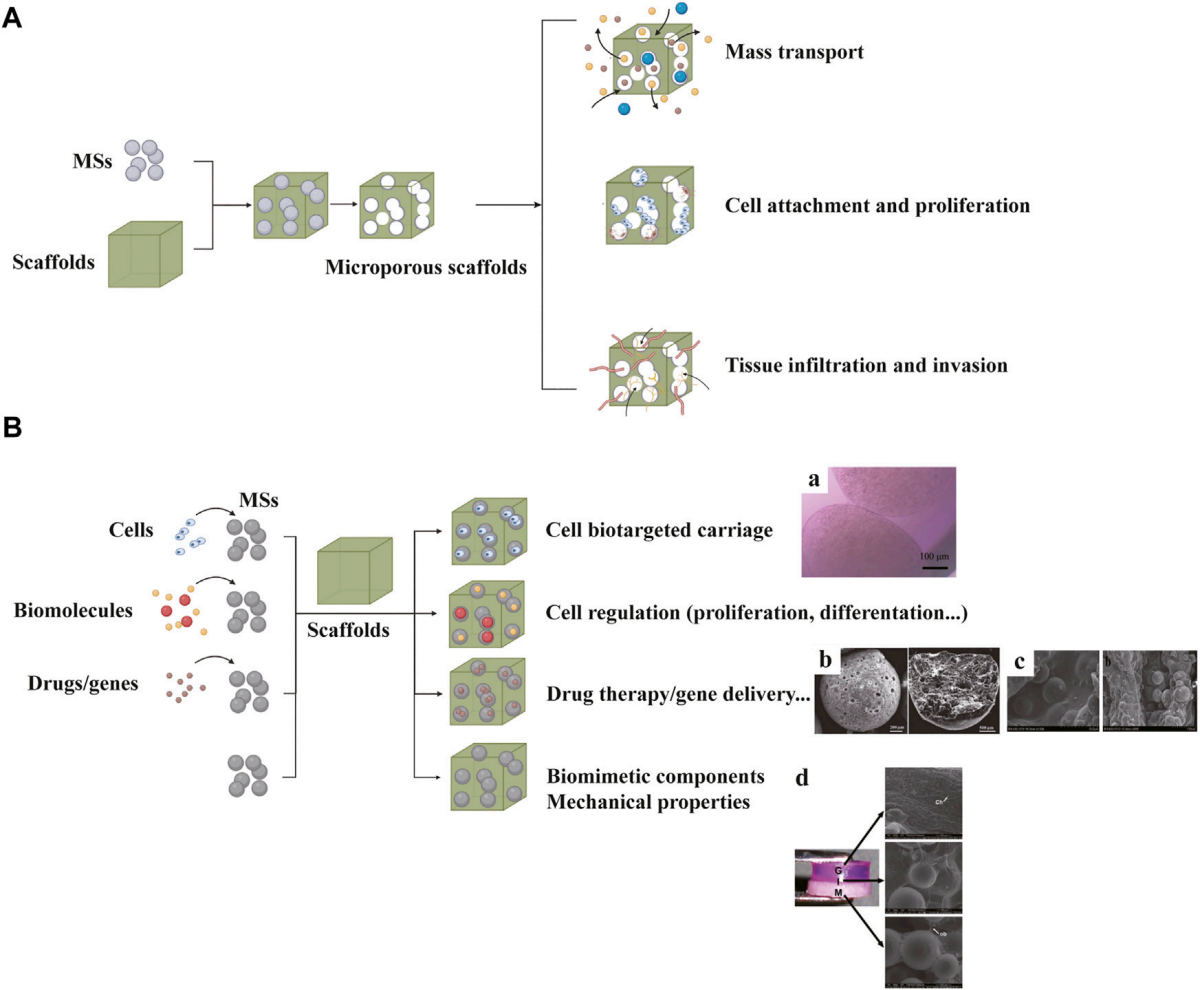
Microsphere (MS) scaffolds can be classified into MS-leached scaffolds **(A)** and MS-incorporated scaffolds **(B)**. Chondrocyte-encapsulated MSs can provide protection and achieve targeted carriage **(B: a)**. MS scaffolds loaded with vancomycin hydrochloride (VH) can reduce inflammation after implantation **(B: b)**. MS scaffolds loaded with OIC-A006 **(B: c)**. Multi-layered scaffold consisting of pre-integrated hydrogel (G), hydrogel + microsphere interface (I) and microsphere (M) bone layers **(B: d)**. **(B-a)** Adapted with permission from ref (Bozkurt et al., 2019). Copyright 2019 by BioMed Central Ltd. **(B-b)** Adapted with permission from ref (He et al., 2021). Copyright 2020 by Elsevier Ltd. **(B-c)** Adapted with permission from ref (Lin et al., 2016). Copyright 2016 by Taylor and Francis Group. **(B-d)** Adapted with permission from ref (Jiang et al., 2010). Copyright 2010 by Biomedical Engineering Society.

As an important candidate as building blocks of scaffolds, microspheres (MSs) show advantages in the following respects: biomimetic structural support, biological regulation, controlled release of biomolecules and drugs, cell delivery *in vivo* and large scale production (He et al., 2020). According to the purpose, scaffolds loaded with MSs come in two types: MS-leached scaffolds and MS-incorporated scaffolds (Figure 6).

In the former type, MSs serve as porogens to produce homeostatic and regular porous structure. Several studies have reported better cell infiltration and tissue invasion based on MS-leached scaffolds (Zaborowska et al., 2010; Huebsch et al., 2015). In the latter type, MSs are used to modify sophisticated biological functions, thereby showing promise for development of osteochondral tissue engineering. MSs based on hydroxyapatite, calcium carbonate, chitosan and PLGA etc. have been reported to use for scaffold fabrications to improve mechanical strength and bioactivity (Shalumon et al., 2016). Cell-encapsulated MSs can protect the inner cells and allow bidirectional substance exchange (Bozkurt et al., 2019). Moreover, MSs loaded with drugs or biomolecules can exert therapeutic effect and regulate cell metabolism (Dormer et al., 2010). Reyes R et al. incorporated MSs loaded with TGF-β1 and BMP-2 into a bilayered scaffold to induce chondrogenic and osteogenic differentiation, separately (Reyes et al., 2014). The microsphere scaffold was reported to induce a high degree of tissue repair based on histological evidence. In addition, one study encapsulated 45S5 bioactive glass particles (BG) in PLGA MSs, which facilitated mineral formation in the interface and bone regions (Jiang et al., 2010). Mohan N et al. fabricated PLGA-CS-NaHCO_3_ as chondrogenic MSs and PLGA-β-TCP as osteogenic MSs to produce a gradient scaffold (Mohan et al., 2015). Recently, various studies have reported the utility of drug or gene delivery MSs for effective osteochondral tissue regeneration (Lin et al., 2016; Madry et al., 2020; He et al., 2021).

### Metal Scaffolds

To date, limited studies on metal scaffolds have been reported. Mrosek EH et al. used trabecular metal/periosteal graft (TMPG) biocomposites to treat osteochondral defects in a sheep model (Mrosek et al., 2016). The metal scaffolds showed excellent bony ingrowth and integration, but failed to promote satisfactory neo-cartilage formation, unfortunately. Another metal worth mentioning is titanium (Ti), a highly corrosion resistant and biocompatible material with superior mechanical properties (Nover et al., 2015). Given the ability to promote cartilage growth and allow bone tissue ingrowth, porous titanium has been proposed as a viable bone-like base material for osteochondral tissue engineering. The mechanical support of subchondral bone has been reported as an essential part in articular cartilage protection (Hu et al., 2021). Therefore, metallic materials have been employed in OC tissue repair as subchondral bone phase. Duan X et al. fabricated a biphasic scaffold composed of type I collagen (Col)/glycosaminoglycan (GAGs) as chondral phase and porous titanium as subchondral phase (Duan et al., 2013). Sing SL et al. combined the two metals mentioned above to develop a biphasic metal scaffold for osteochondral defect repair (Sing et al., 2017). The titanium-tantalum (TiTa)/type I collagen scaffold demonstrated continuous interface between the two phases, the biological functions of which need to be further evaluated. The poor biodegradability and unsatisfactory performance in mimicking articular cartilage tissue have been the major obstacles to widespread application of metals (Díaz Lantada et al., 2016).

### Composite Scaffolds

**FIGURE 7:**
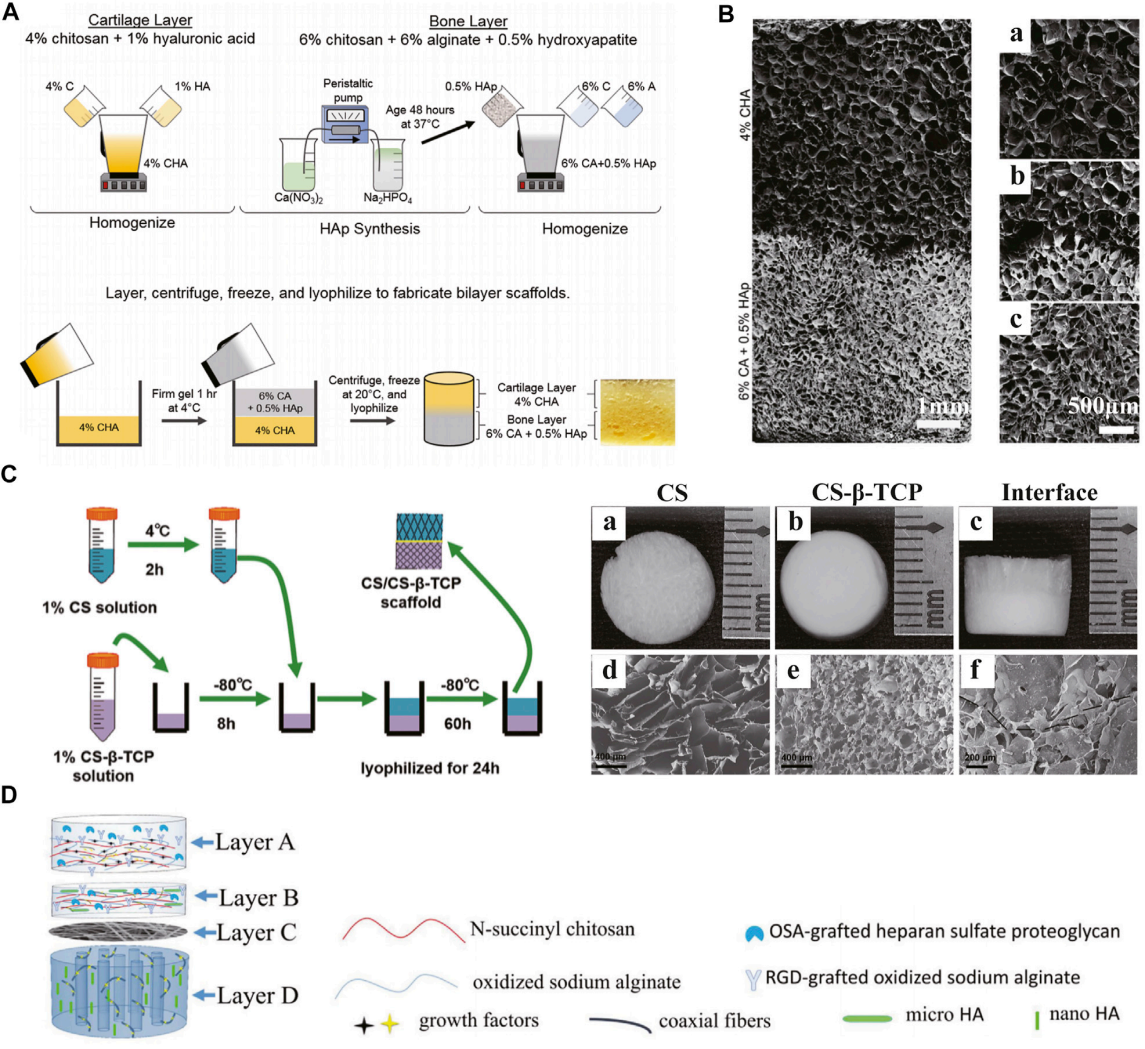
Composite scaffolds. The fabrication process **(A)** and SEM images **(B)** of a bilayered composite scaffold consisting of 4% CHA cartilage layer **(B: a)** and 6% CA + 0.5%HAp bone layer **(B: c)** with an integrated interface **(B: b)**. A bilayered CS/CS-β-TCP scaffold **(C)** was prepared and observed by gross appearance **(C: a–c)** and SEM **(C: d–f)**. A composite scaffold with four functional layers improved the integration of implants with host tissues **(D)**. **(A and B)** Adapted with permission from ref (Erickson et al., 2019). Copyright 2019 by Springer Science + Business Media LLC. **(C)** Adapted with permission from ref (Xu et al., 2021). Copyright 2021 by Mary Ann Liebert Inc. **(D)** Adapted with permission from ref (Chen et al., 2018). Copyright 2018 by Elsevier B.V.

The use of composite scaffolds in tissue engineering can integrate the advantages of polymers and ceramics. Various bioceramics have been used for scaffold fabrication, mainly summarized into three categories including bioinert materials (e.g., alumina and zirconia), semi-inert surface reactive materials (e.g., bioactive glass and dense hydroxyapatite) and biodegradable materials (e.g., calcium phosphate and tricalcium phosphate) (Pina et al., 2018). Unlike polymers, ceramics have better mechanical stiffness and corrosion resistance. However, several inherent features, such as fragility, inelasticity and difficult processing, limit their applications. Therefore, composite scaffolds made of polymers and ceramics perform better in mechanical properties and degradation behaviors. Erickson AE et al. presented a bilayered scaffold composed of two areas (Erickson et al., 2019) (Figures 7A,B). The cartilage and bone layers contain chitosan/hyaluronic acid (HA) and chitosan/alginate/hydroxyapatite nanorod (HAp), respectively. By the thermally-induced phase separation process, a gradient transition zone between the two layers was established, improving the whole stability. A bilayered chitosan/chitosan-β-tricalcium phosphate (CS/CS-β-TCP) composite scaffold was fabricated and evaluated in a rat model (Xu et al., 2021) (Figure 7C). Cells seeded in both layers maintained excellent viability and differentiated into chondrogenic and osteogenic lineages, respectively. As one of the major projects in designing OC tissue engineering scaffolds, the bone-cartilage interface can regulate the mechanical properties of the composite structure and enhance the integration as a connecting medium. Meanwhile, the interface can inhibit unexpected infiltration and provide independent microenvironment for differentiation. For instance, a four-layered composite scaffold was designed to improve the interfacial bonding between the newly formed tissues and the integration of implants with host tissues (Figure 7D). In the study by Khader A et al., fibrous zinc oxide (ZnO)/PCL composite scaffolds were fabricated and evaluated *in vitro* (Khader and Arinzeh, 2020). Different percentage ZnO composite scaffolds demonstrated chondrogenic and osteogenic differentiations in different degrees. Bioactive glass (BG) is another important material to produce polymer-bioceramic composite scaffolds (Yao et al., 2014). Due to its osteogenic potential, BG has a remarkable application prospect in osteochondral defect repair. All these studies emphasize the value of composite scaffolds in effective osteochondral tissue engineering. At present, the main challenges in designing this type of scaffolds lie in the balance among stability, mechanical stiffness, biocompatibility, bioactivity and degradability.

### Extracellular Matrix-Based Scaffolds

In consideration of the difficulty in mimicking comparable components and complicated architecture of native extracellular matrix (ECM), decellularization of specific tissues, by removing cells and genetic molecules and maintaining biochemical and biomechanical properties, is a potential candidate for scaffold fabrication in osteochondral tissue engineering (Hussey et al., 2018; Yin et al., 2018). Decellularized matrix derived from different tissues, such as urinary bladder, pericardium, dermis and cartilage etc., has been developed and investigated *in vitro* and *in vivo* (Singh et al., 2018; Bock et al., 2020). However, decellularized extracellular matrix (dECM), processed to serve as a whole scaffold, has several limitations. Due to the differences in tissue sources, separation and processing techniques and sterilization methods, decellularized scaffolds obtained have certain differences in structures and functions, limiting the reproducibility. Furthermore, unseeded scaffolds promote the natural rebuilding of host tissues, possibly resulting in poor tissue replacement and undesired inogenesis. Efforts should be made to control the potential negative outcomes. Besides, the recellularization of dECM-based scaffolds to mimic native cell distribution is another challenge, considering cell type and concentration, seeding routes and methods and bioreaction characteristics (Hussey et al., 2018). Recently, studies on exosomes possibly pave a new way for OC tissue engineering strategies. Jiang S et al. applied cartilage dECM in combination with intra-articular injection of MSC derived exosomes for osteochondral tissue regeneration (Jiang et al., 2021).

## Clinical Products

To date, several synthetic scaffolds applied to osteochondral lesions have been translated into commercial products, some of which have been approved in the European Union, including TruFit™, MaioRegen™, ChondroMimetic^®^, BioMatrix CRD and Agili-C™ (Wang et al., 2020; D'Ambrosi et al., 2019; Getgood et al., 2012; Kon et al., 2016) (Table 3). Although these bilayered and multilayered scaffolds have reported considerable improvement in clinical symptoms and activity quality, several problems in clinical trials remain to be solved, such as the delamination of scaffolds, the incomplete integration with native tissue, the unsatisfactory quality of regenerated tissue, knee discomfort and the risk of reoperation etc. (Hindle et al., 2014; Brix et al., 2016; Farr et al., 2016; Azam et al., 2018). Therefore, further efforts are needed to illustrate the relationship between these problems and scaffold design strategies for volume production and clinical application.

**TABLE 3:** Hierarchical commercial products for OC tissue regeneration approved in the European Union.

Product name	Company	Classification	Materials	Figures
TruFit™	Smith and Nephew	Biphasic	PLGA-PGA (75:25), calcium sulfate	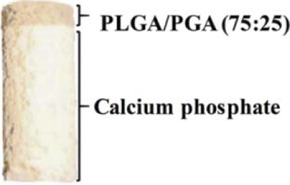
MaioRegen™	Finceramica	Triphasic	Type I collagen, magnesium-enriched hydroxyapatite	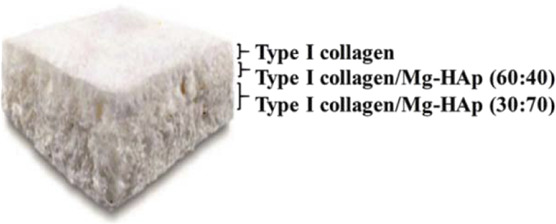
ChondroMimetic^®^	TiGenix	Biphasic	Type II collagen, type I collagen, chondroitin sulfate, calcium phosphate	—
Agili-C™	CartiHeal Ltd	Biphasic	Hyaluronic acid, crystalline aragonite	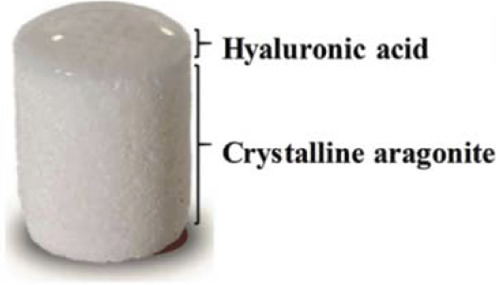
BioMatrix CRD	Kensey Nash	Biphasic	Bovine collagen, β-TCP, PLA	—

Abbreviations: PLGA, poly(lactic-co-glycolic) acid; PGA, polyglycolic acid; PLA, polylactic acid. Figures were reprinted from ref (Zhang B et al., 2020) with permission from The Royal Society of Chemistry, Copyright 2020.

## Current Challenges and Future Prospects

**FIGURE 8:**
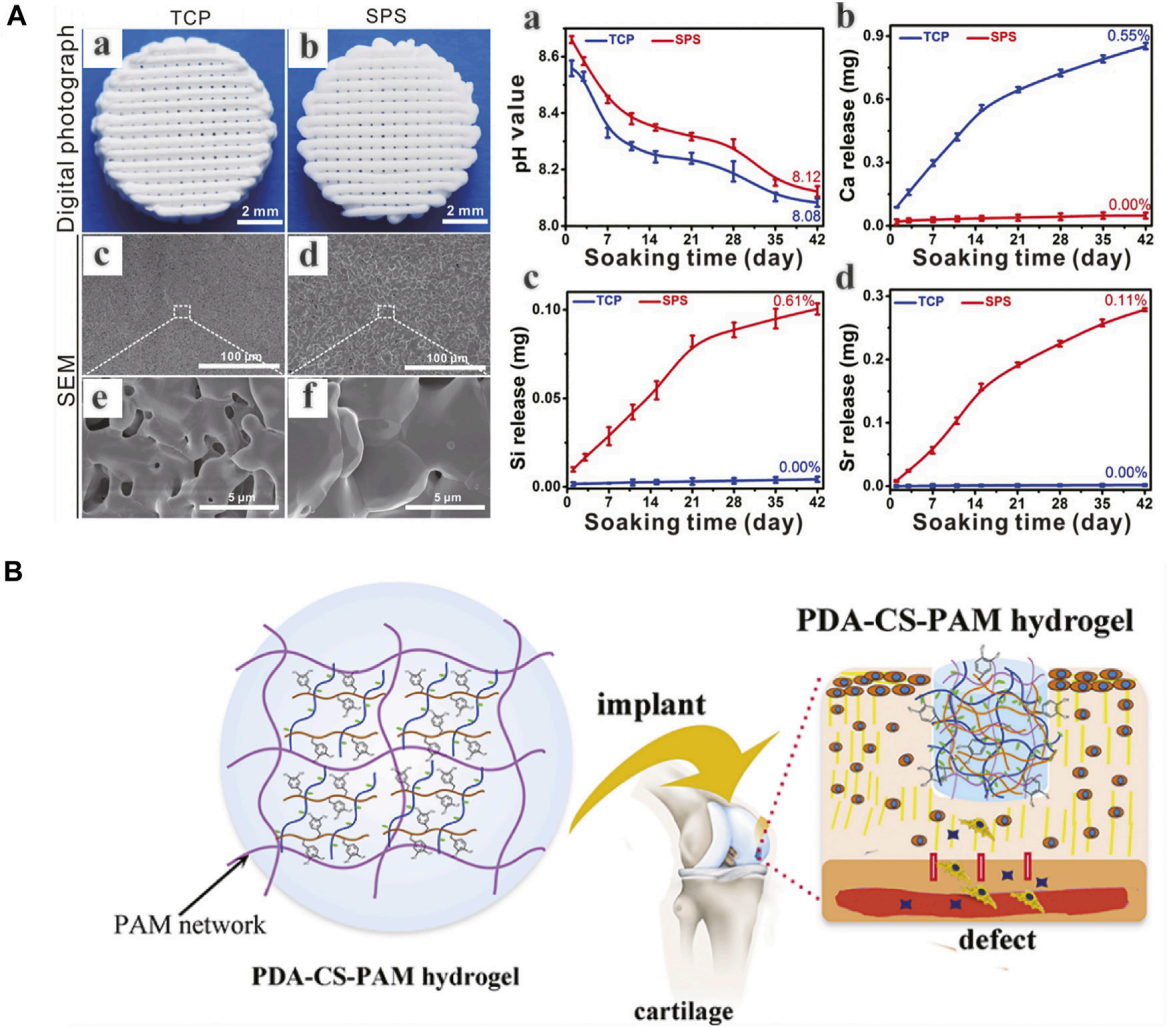
**(A)** 3D-printed Sr_5_(PO_4_)_2_SiO_4_ (SPS) bioactive ceramic scaffolds can promote osteochondral defect reconstruction possibly by releasing Sr and Si ions (a-d). **(B)** A polydopamine−chondroitin sulfate−polyacrylamide (PDA−CS−PAM) hydrogel with tissue adhesiveness and super mechanical properties for cartilage regeneration. **(A)** Adapted with permission from ref (Deng et al., 2018). Copyright 2018 by Ivyspring International Publisher. **(B)** Adapted with permission from ref (Han et al., 2018). Copyright 2018 by American Chemical Society.

Due to the native complexity of OC tissue in hierarchical structure and biological functions, there are still several drawbacks and challenges remaining to be overcome, such as scaffold material-cell interactions, controlled cellular proliferation and differentiation, vascularization and long-term survival of the regenerated tissue etc. Chondrocytes are decisive to the maintenance of the structure and functions of cartilage tissue and can be seeded into scaffolds to produce cartilaginous extracellular matrix (ECM) (Malda et al., 2019). Considering the limitations of chondrocytes, such as isolation difficulty, low proliferation capacity, unexpected dedifferentiation and phenotypic instability, stem cells have offered a new kind of way for OC tissue regeneration. The respective chondrogenic and osteoblastic differentiation of stem cells depends on independent microenvironments, with the assistance of biological or physical stimulations or combined applications (Xuan et al., 2020; Paggi et al., 2020). The spatio-temporal control of stem cell differentiation is an important direction for further research. Due to the low survival rate of cells and instability of bioactive molecules, cells or growth factors incorporated scaffolds are difficult to store and transport, which is an obstacle to commercialization of bioactive scaffolds (Deng et al., 2018). Scaffolds in combination with multiple bioactive ions provide potential directions (Deng et al., 2018) (Figure 8A). Despite the hypothesis that MSCs can differentiate into chondrocytes to regenerate damaged tissue, their secretory functions have attracted more attention recently in tissue repair. Exosomes are possibly candidate for OC tissue engineering (Zhang et al., 2018). Besides, since cells and fibers in native OC tissue have gradient arrangements, the effects of filament orientations and constructs on cellular functions and tissue regeneration should be investigated (Zhang B et al., 2020). Moreover, the integration of scaffolds and regenerating tissue to surrounding targeted tissue is another consistent improving direction, determining the biomechanical properties of the composite structure (F. Zhou et al., 2018). Several strategies, including tissue adhesive and chemical modification, have been applied to enhance integration (Han et al., 2018; Zhang J et al., 2020) (Figure 8B). Despite the comprehensive understanding of the composition and structure of native OC tissue, the reconstruction of its anatomic and biomechanical gradients still remains difficult, which affects the long-term survival of newly formed tissue during joint movements. In comparison to the early developed monophasic scaffolds, biphasic scaffolds can achieve the regeneration of two individual layers, respectively, and triphasic scaffolds can better resemble the OC tissue stratification by taking intermediate CC layer into account. And recently proposed gradient scaffolds, whether in multiphasic or continuous forms, can better imitate the native biochemical components and architectural properties of OC tissue in a more superior way (Wei and Dai, 2021). As a potential direction in future studies, the development of sophisticated gradient OC tissue engineering scaffolds needs more exploration with the assistance of evolving biomaterials and fabrication techniques. Finally, good manufacturing practices and regulatory issues before and after clinical approval are critical to produce safe and efficacious commercial products (Zhou et al., 2020).

## Conclusion

Over the past few decades, osteochondral tissue engineering strategies based on scaffolds have achieved great progress. However, several problems still remain in the restoration of anatomical, biochemical and biomechanical stratification, including the efficiency of cells and bioactive molecules, the integration to adjacent tissues, the spatiotemporal control of physicochemical properties and cellular behaviors and the survival of regenerated tissue etc. In spite of all these challenges, we are optimistic that the development of closely related disciplines and further researches will gradually provide more opportunities for osteochondral tissue engineering for the foreseeable future.

## Supplementary Material

Figure S1 The osteochondral (OC) tissue was observed with Safranin-O/Fast green staining in longitudinal (A) and cross (B) sections. And 3D-model of hyaline cartilage, calcified cartilage zone and subchondral bone was reconstructed. The lower interface of tidemark (C), the upper interface of tidemark (D), the upper interface of cement line (E) and the lower interface of cement line (F) were shown respectively. Scale bar: 200 μm. HC, hyaline cartilage; CCZ, calcified cartilage zone; SB, subchondral bone; TM, tidemark; CL, cement line. Adapted with permission from ref (Wang F et al., 2009). Copyright 2009 by Elsevier Inc.
