# Supplementary material for: Scaffold-Based Tissue Engineering Strategies for Osteochondral Repair
Source: Front Bioeng Biotechnol. 2022 Jan 11;9:812383. doi: 10.3389/fbioe.2021.812383 (PMC8787149; doi:10.3389/fbioe.2021.812383)
Supplement: Supplementary file 1 [file Table1.docx]

**Table S1.** Different types of scaffolds in osteochondral tissue engineering.

| **Classification** | | **Study** | **In vitro/vivo** | **Scaffold design** | **Cells** | | **Findings** |
| --- | --- | --- | --- | --- | --- | --- | --- |
|  |  |  |  |  | **Cartilage compartment** | **Subchondral bone compartment** |  |
| **Porous** | | Duan P et al., 2014, China | Rabbit | PLGA scaffold | BMSCs | — | - Cell-seeded scaffold with pore size of 100–200μm for chondral layer and 300–450μm for osseous layer facilitated the best tissue repair |
|  |  | Schaefer D et al., 2000, America | In vitro | Fibrous, non-woven PGA meshes +  PLGA/PEG foams | Chondrocytes | Periosteal cells | - Mature bone-like constructs to achieve mineralization in combination with immature cartilaginous constructs to promote integration at the tissue interface |
|  |  | Seo JP et al., 2013, Japan | Horses | PRP/acidic GT sponge +  BMP-2/basic GT sponge | Chondrocytes + BMSCs | BMSCs | - PRP/BMP-2/GT scaffolds showed more cartilage-like tissue with no remaining implant materials and no evidence of infection, adhesions or synovial proliferation |
|  |  | Dresing I et al., 2014, Switzerland | Rabbits | PUR + nHA/PUR scaffold,  PUR salt leaching +  electrospun PUR +  nHA/PUR salt leaching | — | — | - An elastomeric PUR scaffold press-fitted into the defect provided a stable matrix - No clear advantage for tissue healing, unfortunately |
|  |  | Jia S et al., 2018, China | Goats | Oriented ACECM-derived cartilage layer +  intermediate compact interfacial layer +  3DP porous PLGA/TCP | — | — | - MLS provided a suitable spatial structure and a template to guide hyaline cartilage, calcified cartilage and subchondral bone growth - The compact interfacial layer developed a smooth osteochondral interface with an integrated tidemark |
|  |  | Jiang Y et al., 2013, China | Rabbits | PVP-I loaded  bilayer collagen scaffold | — | — | - PVP-I incorporated scaffolds showed faster subchondral bone regeneration and remodeling |
|  |  | Kandel RA et al., 2006, Canada | Sheep | CRP | Chondrocytes | — | - Evidence of cartilage maturation, e.g., the increase in the elastic equilibrium modulus with time |
|  |  | Re'em T et al., 2012, Germany | Rabbit | 0.1% alginate-sulfate (w/v), 1% alginate  (w/v) and 0.18% (w/v) D-gluconic acid/hemicalcium salt, loaded with TGF-β1 or  BMP-4 | BMSCs | BMSCs | - Cartilage ECM proteoglycans with no mineralization in the top layer and newly formed woven bone underneath |
|  |  | Zhang YT et al., 2017, China | Rabbits | Bilayered PLGA / autologous PRP scaffold | — | — | - PLGA/PRP scaffolds showed regenerated tissue integrated well with the adjacent cartilage and more smooth surfaces - Higher collagen type II and aggrecan expression level |
|  |  | Cao T et al., 2003, Singapore | In vitro | FDM-fabricated PCL scaffold, three-angle lay-down  pattern (0/60/120°) | Chondrocytes | Iliac crest MSCs | - A mixture of cell types at the interface - Dense and mineralized ECM deposited in bone compartment - Decreased osteocalcin expression in the coculture situation |
|  |  | Wu X et al., 2021, China | Rabbits | Bilayer silk scaffold  TGF-β3-loaded Sil-MA hydrogel (10%D/S)+  BMP-2-loaded porous layer (30%P/S) | — | — | - The effect of the Sil-MA hydrogel as a marginal sealant on the integration of the cartilage layer and rapid cartilage formation - The biomimetic design of the cartilage layer benefited chondral regeneration - BMP-2-loaded porous structure promoted rapid bone formation |
|  | | Levingstone TJ et al., 2016, Ireland | Rabbits | 0.125% (w/v) Col1/0.375% (w/v) Col2/0.05% (w/v) HyA +  0.5% (w/v) Col1/0.5% (w/v) Col2/0.2 % (w/v) HA +  0.5% (w/v) Col1/1% (w/v) HA | — | — | - Improved integration with native tissue with less obvious central depression and fissuring of the surface |
| **Hydrogel** | | Liao J et al., 2017, China | Rats/Rabbits | CAN-PAC hydrogel  CSMA/NIPAm +  PECDA/AAm/PEGDA | Chondrocytes | Osteoblasts | - New translucent cartilage and repaired subchondral bone - The regeneration of cartilage with similar mechanical properties as normal cartilage |
|  |  | Dehghani Nazhvani F et al., 2021, Iran | Rabbits | Chitosan-based hydrogel scaffolds with CNF or HA | BFPSCs | BFPSCs | - Hypoxic preconditioned scaffolds showed a faster healing trend, thicker cartilage, more new ECM formation and proteoglycans expression - More new ECM deposition in BFPSCs-seeded groups |
|  |  | Pirosa A et al., 2021, America | In vitro | Solid gelMA hydrogel +  wet-spun PCL +  PCL/HA scaffold | hMSCs:HUVECs (1:4 ratio) | hMSCs:HUVECs  (1:4 ratio) | - Vascularized osteochondral interface tissue using a dual chamber microphysiological system bioreactor for induced differentiation of each compartment |
|  |  | Boyer C et al., 2020, France | Dogs | Silanised hydroxypropymethyl cellulose (Si-HPMC)/chitosan hydrogel | ADSCs | ADSCs | - The injectable and self-hardening hydrogel promoted cell survival and activity. - Osteochondral defect regeneration in a dog model after implantation with or without ADSCs. |
| **Fibrous** | | Hild N et al., 2011, Switzerland | In vitro | Bilayered membranes,  PLGA  +calcium phosphate (CaP)/collagen (Col)/PLGA nanofibers | hMSCs | hMSCs | - Better cell proliferation and differentiation into the osteogenic lineage. - Biomineralization was observed preferably on the CaP/Col/PLGA side. |
|  |  | Liu S et al., 2015, China | Rabbits | Nanofiber yarn-collagen type I/hyaluronate hybrid (Yarn-CH)/  TCP biphasic scaffold | BMSCs | BMSCs | - Differentiated BMSCs/Yarn-CH/TCP scaffolds showed an almost smooth articulating surface - Good integrity of the cartilage at the host cartilage–implant interface |
|  |  | Zhang S et al., 2013, China | Rabbits | Bilayered microporous scaffolds,  Collagen/electrospun poly-L-lactic acid nanofibers (COL-nanofibers) | — | — | - The COL-nanofiber scaffold exhibited rapid osteogenic differentiation and better cartilage formation. |
|  |  | Bhardwaj G et al., 2016, Saudi Arabia | In vitro | XanoMatrix nanofibrous scaffolds,  Polyethylene terephthalate/cellulose acetate | Chondrocytes | Chondrocytes | - Greater hydrophobicity, 3D surface area and greater tensile strength. - The nanofibrous scaffolds promoted chondrocyte growth and proliferation. |
|  |  | Elangomannan S et al., 2017, India | Rats | CNF/PCL/mineralized hydroxyapatite (M-HAP) nanofibrous scaffolds | — | — | - Higher cell viability and soft fibrous tissue growth without inflammation. |
|  |  | Jeon JE et al., 2014, Australia | Rats | Alginate (S) +  alginate (MD) +  PCL-FDM +  PCL scaffold | Chondrocytes | Osteoblasts | - Limited mineralization in the PCL compartment with or without osteoblasts pre-seeded - Limited blood vessel network within the osseous construct while many within the bovine bone - Fibrous tissue infiltration with macrophage-like multinucleated cells indicated possible inflammatory responses |
| **Microsphere** | | Shalumon KT et al., 2016, China | Nude mice | Microsphere-based bilayered scaffolds,  PLGA microspheres  +10% nanohydroxyapatite  (nHAP)-incorporated PLGA (PLGA/nHAP) microspheres | Chondrocytes | BMSCs | - The co-cultured scaffolds showed cell proliferation and tissue development with gross morphological and electron microscopic evidence in an ectopic osteochondral defect model. |
|  |  | Reyes R et al., 2014, Spain | Rabbits | Alginate and TGF-β1-loaded microspheres +  porous PLGA cylinder with BMP-2-loaded microspheres | — | — | - Scaffolds loaded with growth factors induced a high degree of repair, with thicker cartilage layer in the center - High dose GF-loaded scaffolds showed clearer signs of repair based on histological scores |
|  |  | Jiang J et al., 2010, America | In vitro | Stratified polymer ceramic-hydrogel scaffold，  Hydrogel (G) +  hydrogel/microspheres (I) +  PLGA-BG microspheres/polymer glass (M) | Chondrocytes | Osteoblasts | - PLGA-BG microspheres facilitated mineral formation - Proteoglycan-rich matrix in the cartilage region while mineralized collagen-rich matrix in the bone region - The effects of chondrocyte density on matrix deposition and mechanical properties |
|  |  | Mohan N et al., 2015, America | Sheep | Microsphere-based gradient scaffolds (+TGF-β3/IGF),  PLGA-Chondroitin sulfate A sodium salt (CS)-NaHCO_3_ microspheres  +PLGA-β-TCP microspheres | — | — | - The CS/β-TCP gradient scaffolds regenerated cartilage similar to native tissue in view of gross structure and cell morphology. |
|  |  | He J et al., 2021, China | In vitro | Chitosan (CS)-coated polytrimethylene carbonate (PTMC)/polylactic acid (PLLA)/oleic acid-modified hydroxyapatite (OA-HA)/vancomycin hydrochloride (VH) microsphere scaffolds | osteoblasts | osteoblasts | - The microsphere scaffold provided a suitable surface for osteoblast adhesion. - The release of OA-HA promoted osteogenic proliferation. |
|  |  | Lin L et al., 2016, China | Rats | PLGA/β-CPT scaffolds with different amounts of OIC-A006 microspheres | — | — | - Low concentration load OIC-A006 microspheres can probably promote bone healing. |
|  |  | Han F et al., 2014, China | Rabbits | Graded CS-GMA/Gel-GMA loaded with TGF-β1+  PLGA scaffold loaded with BMP-2 | BMSCs | BMSCs | - TGF-β1/BMP-2 loaded scaffolds showed an integrated cartilage repair surface with subchondral bone formation in the bottom - TGF-β1 promoted hyaline-like cartilage tissue regeneration - BMP-2 stimulated chondrogenic and osteoblastic differentiation and promoted ALP activity and Ca^2+^ content |
| **Metal** | | Mrosek EH et al., 2016, America | Sheep | Trabecular metal/periosteal graft (TMPG) | — | — | - TMPG scaffolds incorporated nicely into the bordering subchondral bone without implant loosening or inflammatory response, but failed to promote satisfactory neo-cartilage formation as TM |
|  |  | Duan X et al., 2013, China | Beagles | Type I collagen (Col)/glycosaminoglycan  (GAGs)-porous titanium biphasic scaffolds (CGT) | BMSCs | BMSCs | - The CGT scaffolds seeded with BMSCs promoted hyaline-like cartilage and subchondral bone formation with no visible inspection. |
|  |  | Sing SL et al., 2017, Singapore | / | Titanium based biphasic scaffolds,  Titanium/type 1 collagen  +titanium-tantalum/type 1 collagen | — | — | - The titanium-tantalum (TiTa)/type I collagen scaffold demonstrated continuous interface between the two phases, the biological functions of which need to be further evaluated. |
| **Composite** | | Erickson AE et al., 2019, America | In vitro | Bilayered composite scaffolds,  Chitosan/hyaluronic acid (HA)  +chitosan/alginate/hydroxyapatite nanorod (HAp) | Human chondrocyte-like cells (SW-1353)/MSCs | Human osteoblast-like cells (MG63) | - The gradient transition zone between the two layers improved the whole stability. - Cell proliferation and migration to the interface. |
|  |  | Xu D et al., 2021, China | Rats | Bilayered composite scaffolds,  Chitosan/chitosan-β-tricalcium phosphate (CS/CS-β-TCP) | — | — | - Chondrocytes and BMSCs seeded separately in the two layers maintained respective differentiation lineages. - The composite scaffolds showed newly formed tissues comprising hyaline-like cartilage and subchondral bone. |
|  |  | Chen T et al., 2018, China | Rabbits | Four-layered composite scaffolds | — | — | - The multilayered composite scaffolds improved the interfacial bonding between the newly formed tissues and the integration of implants with host tissues. |
|  |  | Khader A et al., 2020, Newark | In vitro | Fibrous zinc oxide (ZnO)/PCL composite scaffolds | hMSCs | hMSCs | - Low percentage ZnO composite scaffolds promoted chondrogenic differentiation, while high percentage ones promoted osteogenic differentiation. |
|  |  | Yao Q et al., 2014, China | In vitro | Bilayered 45S5 Bioglass®/chitosan-polycaprolactone scaffolds | Osteoblast-like cells (MG-63) | Osteoblast-like cells (MG-63) | - The coated BG scaffolds demonstrated suitable bioactivity and lower degradation rate. - Both coated and uncoated scaffolds promoted cell adhesion and growth. |
|  |  | Ding C et al., 2013, China | Nude mice | CAD/CAM-fabricated scaffold, PGA/PLA +  PCL/HA | Chondrocytes | BMSCs | - A tissue specific regeneration with cartilage tissue, immature calcified tissue, transitional trabecular bone and hypertrophic chondrocytes |
|  |  | Ribeiro VP et al., 2019, Portugal | In vitro | HRP-SF +  HRP-SF/dTCP | Chondrocytes | Osteoblasts | - A specific stimulation of the chondrogenic and osteogenic cells in the co-culture system - The potential to induce chondrocyte pre-hypertrophy |
|  |  | Natarajan ABM et al., 2021, India | In vitro | Selected PCL/PLGA/CS(65:30:5) +  PCL/PLGA/βTCP(62.5:31.25:6.25) in a honeycomb pattern | — | — | - Higher cellular metabolic activity on CS and βTCP loaded scaffolds - Excellent cell-attachment affable properties - Evaluation of chondrogenesis and osteogenesis respectively - Significant upregulation of chondrocyte/bone marker gene expression |
|  | Kim S H et al., 2015, Korea | | BALB/c mice | Elastic PLCL scaffold +  PLGA/β-TCP scaffold | Chondrocytes | BMSCs | - Improved chondrogenic and osteogenic differentiation - Increased chondral ECM - Presence of calcium phosphates in bone |
|  | Zhu M et al., 2020, China | | Mechanics | Integrated triphasic MBG-alginate scaffold,  SA hydrogel (5 layers)+  SA/MBG compact (2 layers)+  SA/MBG scaffold (8 layers) | — | — | - A tri-layered graded SA/MBG scaffold using 3D printing with improved mechanical interface bonding strengths |
| **Decellularized matrix** | Degirmenci E et al., 2019, Turkey | | Rabbits | Hyalofast® | — | — | - TA improved osteochondral defect repair in combination with acellular scaffolds in terms of the healing time and tissue stability. |
|  | Jiang S et al., 2021, China | | Rabbits/Rats | Acellular cartilage extracellular matrix (ACECM) scaffolds+intra articular injection of human umbilical cord Wharton’s jelly MSC-Exos (hWJMSC-Exos) | — | — | - In vitro, hWJMSC-Exos promoted cell proliferation and migration. - Better osteochondral regeneration and improved microenvironment of the articular cavity. |
|  | Ma N et al., 2017, China | | RCT (Clinical trials) | Biomimetic cartilage extracellular-matrix-oriented scaffolds | Autologous chondrocytes | — | - A completely natural scaffold for the clinical practice in cartilage repair. |
|  | Ye K et al., 2018, Australia | | Rabbits | Acellular dermal matrix (ADM) | — | — | - ADM can promote revascularization, cartilage repair, tissue remodeling and anatomical interface reconstruction. |
