# Supplementary figures and images for: Scaffold-Based Tissue Engineering Strategies for Osteochondral Repair
Source: Front Bioeng Biotechnol. 2022 Jan 11;9:812383. doi: 10.3389/fbioe.2021.812383 (PMC8787149; doi:10.3389/fbioe.2021.812383)

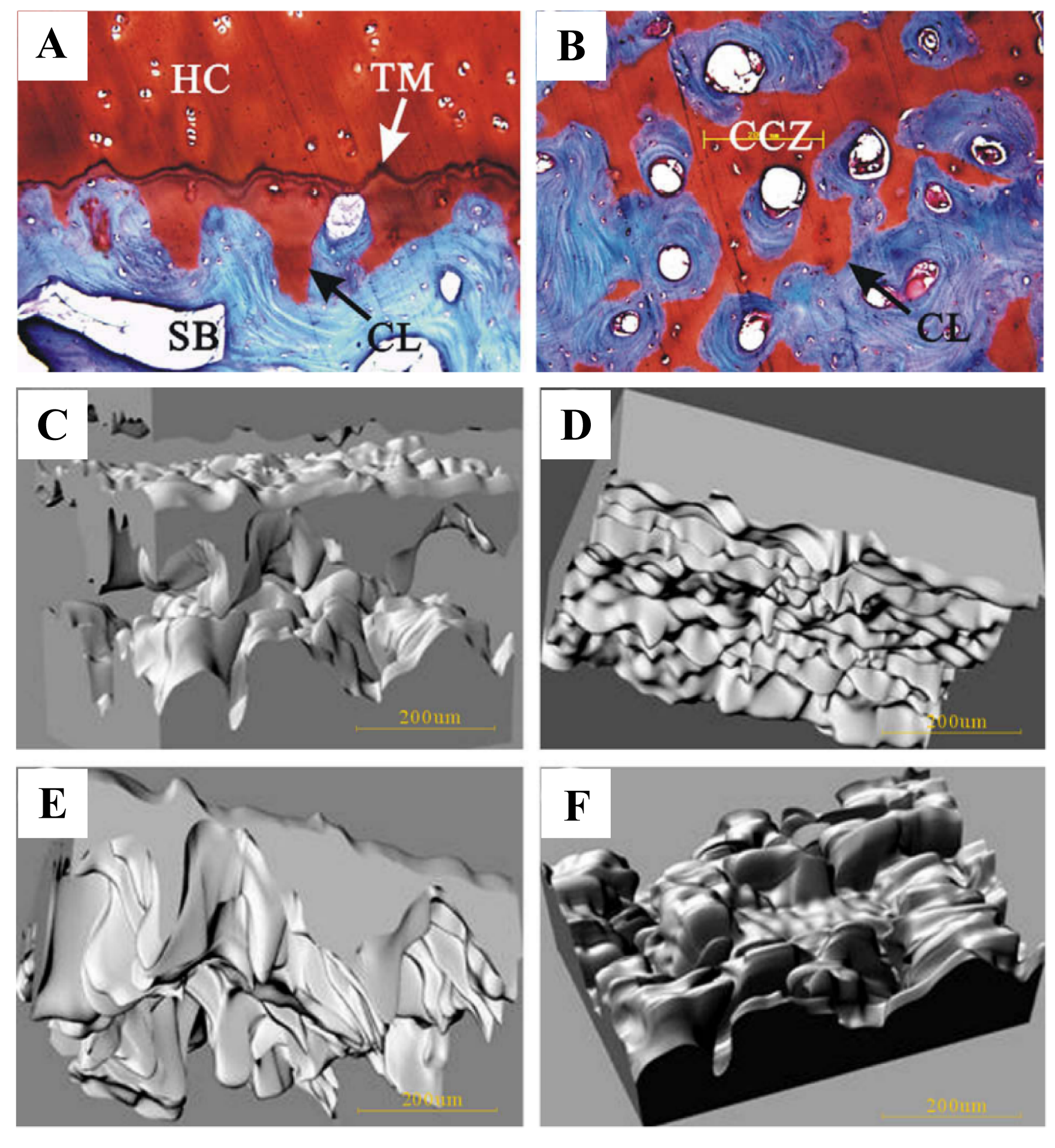

Supplement: Supplementary file 2 [file Image1.TIF]
